# Nanofat-derived stem cells with platelet-rich fibrin improve facial contour remodeling and skin rejuvenation after autologous structural fat transplantation

**DOI:** 10.18632/oncotarget.19721

**Published:** 2017-07-31

**Authors:** Hua Wei, Shi-Xing Gu, Yi-Dan Liang, Zhi-Jie Liang, Hai Chen, Mao-Guang Zhu, Fang-Tian Xu, Ning He, Xiao-Juan Wei, Hong-Mian Li

**Affiliations:** ^1^ Department of Endocrinology, The Affiliated Hospital of Youjiang Medical University for Nationalities, Baise 533000, China; ^2^ Department of Burns & Plastic Surgery, The Affiliated Hospital of Youjiang Medical University for Nationalities, Baise 533000, China; ^3^ Central Laboratory of Medical Science, The Fifth Affiliated Hospital of Guangxi Medical University & The First People’s Hospital of Nanning, Nanning 530022, China; ^4^ Department of Gland Surgery, The Fifth Affiliated Hospital of Guangxi Medical University & The First People’s Hospital of Nanning, Nanning 530022, China; ^5^ Department of Orthopedics, The First Affiliated Hospital of Gannan Medical University, Ganzhou 341000, China; ^6^ Department of Plastic and Aesthetic Surgery, The Fifth Affiliated Hospital of Guangxi Medical University & The First People’s Hospital of Nanning, Nanning 530022, China; ^7^ Department of Urinary Surgery, The Affiliated Hospital of Youjiang Medical University for Nationalities, Baise 533000, China

**Keywords:** nanofat, nanofat-derived stem cells, stromal vascular fraction, platelet-rich fibrin, structural fat transplantation

## Abstract

Traditional autologous fat transplantation is a common surgical procedure for treating facial soft tissue depression and skin aging. However, the transplanted fat is easily absorbed, reducing the long-term efficacy of the procedure. Here, we examined the efficacy of nanofat-assisted autologous fat structural transplantation. Nanofat-derived stem cells (NFSCs) were isolated, mechanically emulsified, cultured, and characterized. Platelet-rich fibrin (PRF) enhanced proliferation and adipogenic differentiation of NFSCs *in vitro*. We then compared 62 test group patients with soft tissue depression or signs of aging who underwent combined nanofat, PRF, and autologous fat structural transplantation to control patients (77 cases) who underwent traditional autologous fat transplantation. Facial soft tissue depression symptoms and skin texture were improved to a greater extent after nanofat transplants than after traditional transplants, and the nanofat group had an overall satisfaction rate above 90%. These data suggest that NFSCs function similarly to mesenchymal stem cells and share many of the biological characteristics of traditional fat stem cell cultures. Transplants that combine newly-isolated nanofat, which has a rich stromal vascular fraction (SVF), with PRF and autologous structural fat granules may therefore be a safe, highly-effective, and long-lasting method for remodeling facial contours and rejuvenating the skin.

## INTRODUCTION

Aside from altering appearance, facial skin and soft tissue aging impacts patients’ psychological well-being and lives to varying degrees [1–3]. There are many causes of depressions and deformities in facial soft tissue. Infection and injury may cause temporal, frontal, and zygomatic soft tissue depression, scars may lead to depression for some patients with cranial dysplasia, and progressive hemifacial atrophy may result for unknown reasons; however, overstrain and deliberate weight loss are the most common causes of soft tissue depression treated via aesthetic surgery [4–6]. Autologous structural fat transplantation is one of the most common methods for correcting facial soft tissue depression. However, 20% to 90% of the fat injected on a given occasion can be easily absorbed, and repeated injections are often necessary to achieve the desired results. Methods for reducing fat absorption and increasing its stability are therefore a focus of current research [7–10].

Due to abundantly available materials, the simplicity of the procedure, low risk of rejection, and its natural appearance, autologous fat transplantation has become the most common method for treating soft tissue defects. However, traditional autologous fat transplantation is associated with lower success rates due to high levels of fat absorption and increased likelihood of unsatisfactory shaping, induration, cysts, and other undesirable effects. Immediate access to the blood supply is crucial for transplant survival in the early stages of recovery. Studies show that an abundance of adipose-derived stem cells (ASCs) in the stromal vascular fraction (SVF) of liposuction aspirates can enhance survival rates by increasing blood supply in fat transplants [11–13]. The clinical success of nanofat in skin rejuvenation procedures, which was first reported in 2013, may also depend on the presence of stem cells [14]. However, additional studies are needed to confirm the efficacy of procedures using nanofat. The aim of this study was to improve nanofat techniques by using a mechanical emulsification method. We generated nanofat particles smaller than 100 μm from activated fat granules by filtering liposuction aspirates through a superfine filter. Isolated culture revealed that many mesenchymal stem cells, designated Nanofat-derived stem cells (NFSCs), were present in the nanofat. Nanofat, which is smaller than traditional fat granules, increases contact between the SVF and large fat granules and strengthens the beneficial effects of SVF in fat transplantation [15–17]. Here, we examined the effects of autologous structural fat transplants mixed with both newly-isolated nanofat and platelet-rich fibrin (PRF) on symptoms of aging, such as facial depression deformity, slack and rough skin, and small wrinkles.

## RESULTS

### Biological characteristics of PRF

**Figure 1:**
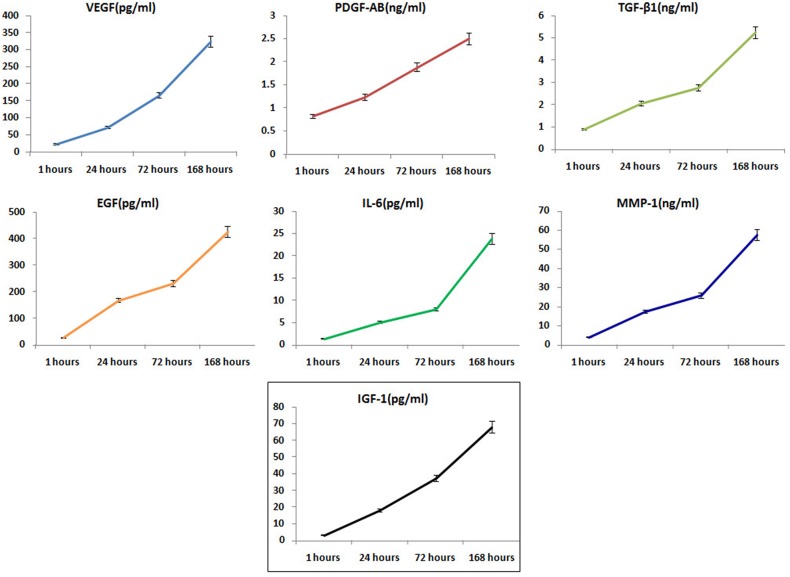
Molecules released from PRF clots over time Release of VEGF, PDGF-AB, TGF-β, EGF, IL-6, IGF-1, and MMP-1 from PRF clots gradually increased in a time-dependent manner (mean ± SD).

The average leukocyte and RBC counts in the PRF-type clots were (5.87 ± 1.45) × 10^3^/μL and (3.14 ± 0.93) × 10^3^/μL, respectively. The mean blood platelet concentration of whole PRF-type clot was (761.5 ± 83.2)× 10^3^/μL (n=62). The vast majority of the PRF clots samples consisted of platelets (>90%). Levels of VEGF, PDGF-AB, TGF-β, EGF, IL-6, IGF-1, and MMP-1 secreted by the PRF clots at different timepoints are shown in Figure 1; VEGF, PDGF-AB, TGF-β, EGF, IL-6, IGF-1 and MMP-1 levels increased in a time-dependent manner (Table 1).

**Table 1:** Levels of molecules release from PRF clots at different timepoints (means ± SD)

Molecule	1 hour	24 hours	72 hours	168 hours
**VEGF (pg/ml)**	23.82±4.03	72.64±10.81	167.43±13.52	325.36±37.64
**PDGF-AB(ng/ml)**	0.83±0.12	1.24±0.15	1.89±0.18	2.51±0.33
**TGF-β1(ng/ml)**	0.91±0.13	2.08±0.25	2.76±0.31	5.26±0.68
**EGF(pg/ml)**	28.45±4.14	169.07±20.32	231.93±31.42	427.13±39.12
**IL-6(pg/ml)**	1.46±0.27	5.11±1.03	8.08±1.14	23.89±3.57
**IGF-1(pg/ml)**	3. 40±0.52	18. 15±3.23	37. 41±5.21	68. 22±8.07
**MMP-1(ng/ml)**	4.22±0.66	17.62±3.08	25.93±3.86	57. 64±6.29

### Biological characteristics of NFSCs

**Figure 2:**
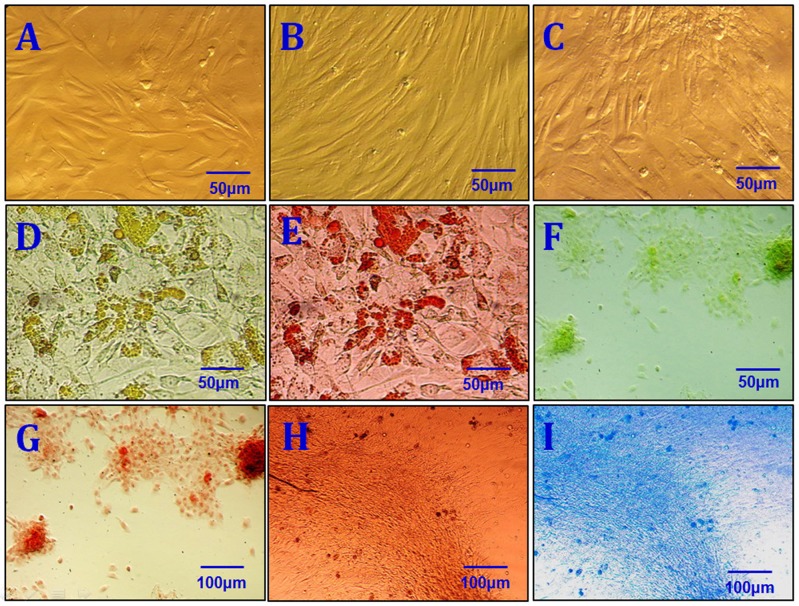
Characterization of NFSCs **(A)** Morphological characterization of primary NFSCs. **(B)** Morphological characterization of P3 NFSCs. **(C)** Morphological characterization of P8 NFSCs. **(D)** Adipogenic induction was performed for 2 weeks before staining. **(E)** Positive oil red O staining after 2 weeks of adipogenic induction. **(F)** Osteogenic induction was performed for 3 weeks before staining. **(G)** Positive alizarin red staining after 3 weeks of osteogenic induction. **(H)** Chondrogenic induction was performed for 3 weeks before staining. **(I)** Positive Alcian blue staining after 2 weeks of chondrogenic induction. Scale bars: 50 μm (A-F); 100 μm (G-I).

**Figure 3:**
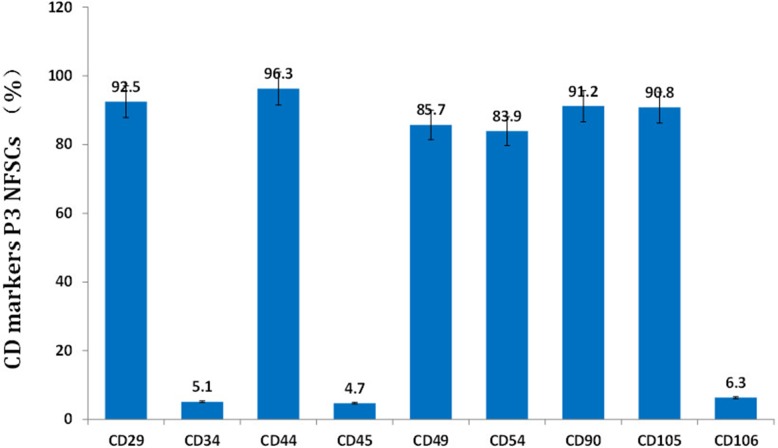
NFSCs surface markers were analyzed using flow cytometry CD29, CD44, CD49d, CD54, CD90, and CD105 expression were high, while CD34, CD45, and CD106 expression were low, in third-passage NFSCs.

After primary cells were cultured for 24 to 48 hours, a few fibroblast-like cells had adhered to the walls; most of the unattached cells were globular or round blood cells. After the first liquid change during the 24-hour culture, most adherent cells were wide and flat fibroblast-like cells; a few triangular or polygonal cells were also observed. As the culture continued, the adherent cells grew in colonies of different sizes. The cells in the colonies were typical fusiform cells. After 10-12 days, the cells had formed an 80%∼90% confluent monolayer. After passaging, most cells were fusiform, and a few were triangular or polygonal (Figure 2A). Passaged cells grew noticeably faster than primary cells and took only 3 days to reach confluence if they were passaged at the proportion of 1:3. Passaged cells were obviously bulged, had fusiform or polygonal shapes, and grew relatively fast. The average cell multiplication time was 40 hours. Cells from all passages were similar in shape to ASCs obtained using the traditional collagenase digestion method in our previous studies [18–22] (Figure 2B and 2C). Third-passage cells underwent 14 days of adipogenic induction, as indicated by a bright lipid droplet stained with red oil O (Figure 2D and 2E). After three weeks, changes were observed in calcified nodules and alizarin red-stained tubercles were visible (Figure 2F and 2G). After 14 days of chondrogenic induction, cells were densely-clustered and had a patchy or tuberculous shape with surrounding cells in a radial pattern. The large tubercles and nearby cells are shown after staining with Alcian Blue (Figure 2H and 2I). Expression of CD29, CD44, CD49d, CD54, CD90, and CD105 was increased, while expression of CD34, CD45, and CD106 was decreased, in third-passage NFASCs (Figure 3).

### Influences of PRF on growth and proliferation in NFSCs

**Figure 4:**
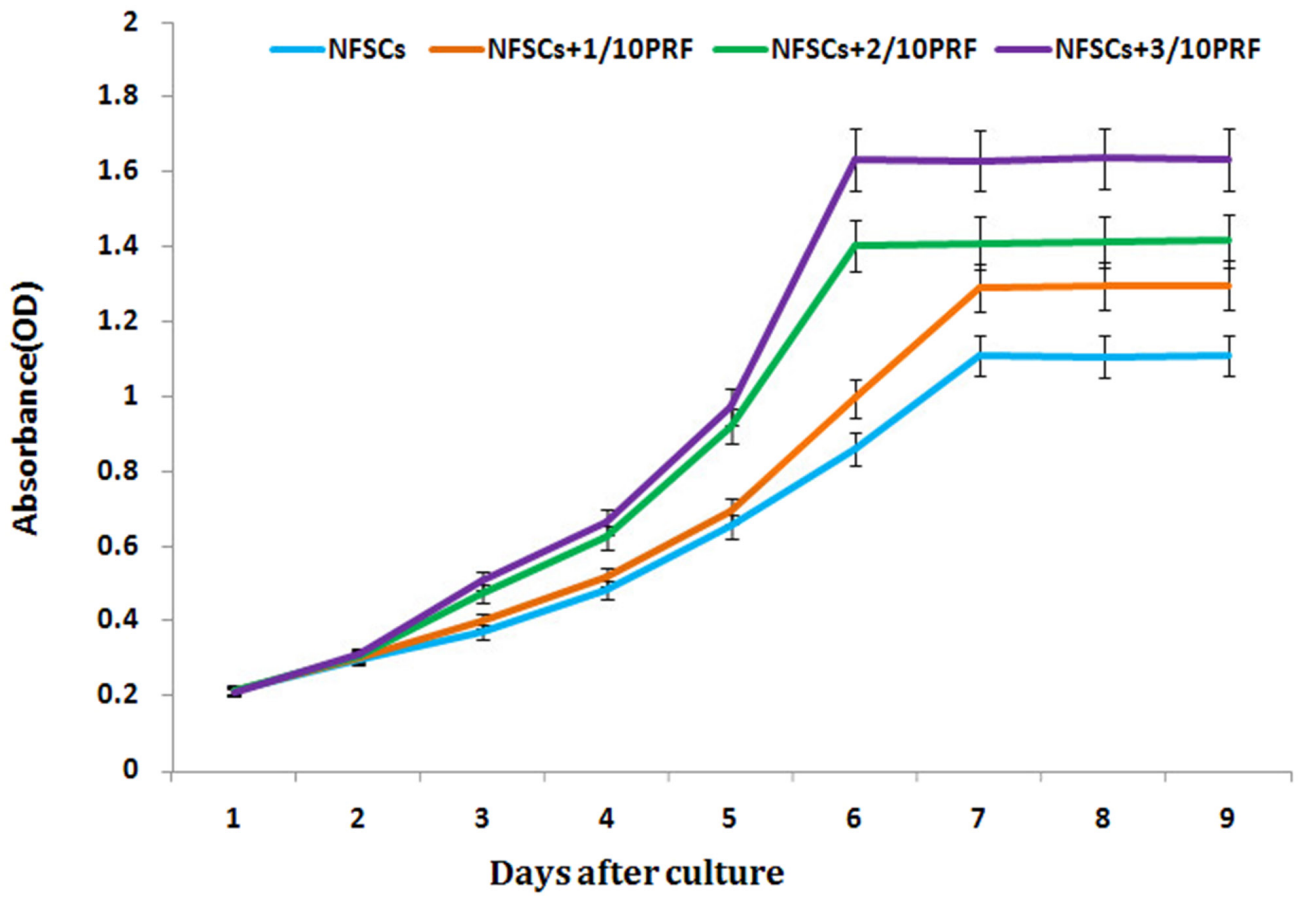
NFSCs proliferation was assessed using a CCK-8 assay Absorbance was higher in the 1/10, 2/10, and 3/10 PRF groups than in the control group (without PRF) at every timepoint from day 3 of the study on. Results are shown as means ± SD, n = 6; *P* < 0.01 using ANOVA.

**Figure 5:**
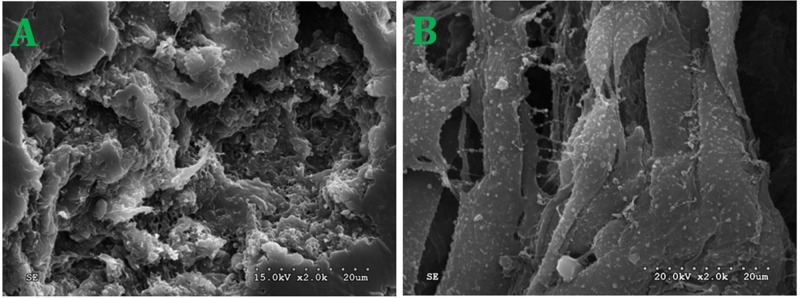
NFSCs morphology and status after co-culture with PRF **(A)** Ultrastructure of the PRF scaffolds. **(B)** NFSCs under SEM after 72 hours of co-culture. SEM×2.0k.

NFSCs were cultured in media with PRF concentrations of 1/10, 2/10, or 3/10. After 1 to 9 days, CCK-8 detection revealed that PRF affected NFSCs proliferation in a dose- and time-dependent manner. The OD values were higher than those of the control group at all timepoints from the third day on for the 2/10 and 3/10 PRF groups and from the fourth day on for the 1/10 PRF group. Cell growth and proliferation reached a plateau after 7 days in the 1/10 PRF group and after 6 days in the 2/10 PRF and 3/10 PRF groups (Figure 4). After 72 hours of co-culture, NFSCs increased markedly in number and began migrating into the pores of the PRF membrane when viewed under scanning electron microscopy. The cells extended pseudopodia and adhered to the well surfaces or pores, aggregated, adhered, and grew on the clot scaffold, and secreted extracellular matrix. These results are consistent with the results of the CCK-8 assays and indicate that the PRF clots promoted cell attachment and growth (Figure 5).

### Concentration-dependent effects of PRF on relative expression of adipogenic genes in human NFSCs

**Figure 6:**
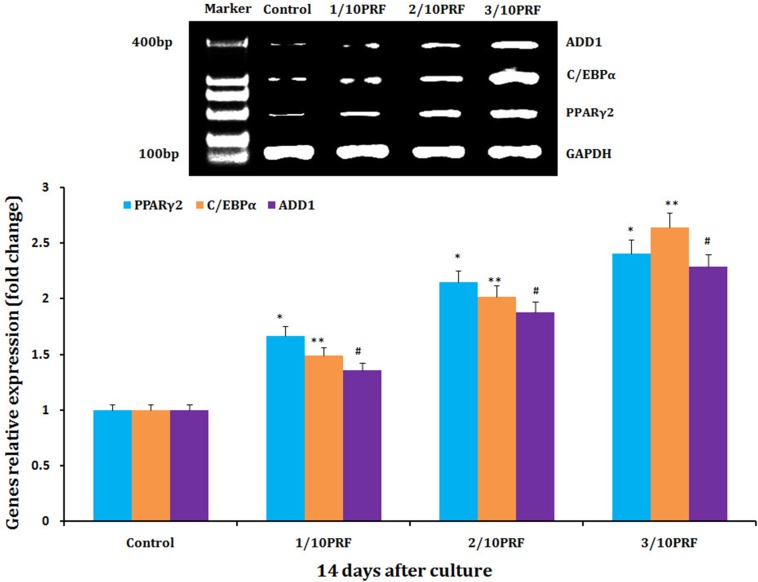
mRNA levels of PPARγ2, C/EBPα and ADD1 mRNA, which are adipogenic marker genes, were much higher in the PRF groups than in the control group after 14 days of culture *P<0.01, **P<0.01, #*P*<0.01.

Human NFSCs were cultured for 14 days in the basal adipogenic induction media combined with PRF concentrations of 1/10, 2/10, and 3/10. qPCR revealed that the PRF concentrations of 1/10, 2/10 and 3/10 increased expression of PPARγ2, C/EBPα, and ADD1 mRNA compared to the control group (the basal adipogenic induction medium group), respectively. At the same time, the mRNA relative expression of PPARγ2, C/EBPα, and ADD1 exhibited a positive concentration-dependent increase on the basis of PRF (Figure 6).

### Analysis of clinical efficacy

**Figure 7:**
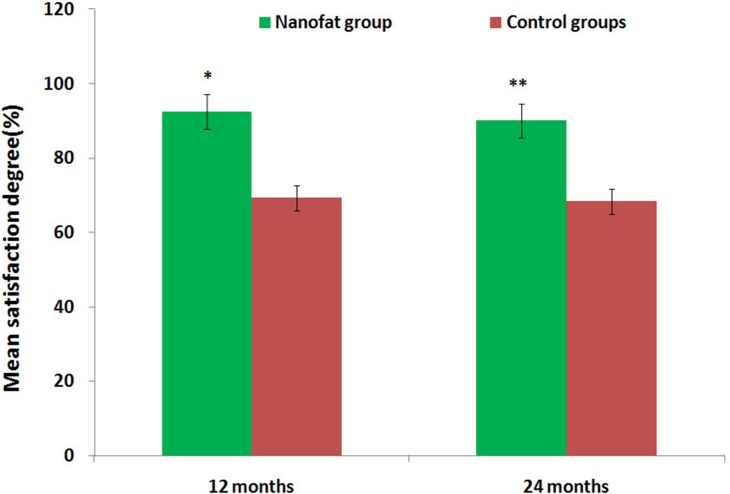
Assessment of mean satisfaction rates for test and control group patients after 12 and 24 months The mean satisfaction rate in test group patients was higher than 90%, while the mean satisfaction rate in control group patients was lower than 70%. *P<0.01, **P<0.01.

Comparisons of preoperative and postoperative photos and relative detection results indicated that all subjects received sufficient filling of soft tissue to improve the appearance of the target area in the first month after the operation. However, the transplanted fat was gradually absorbed over the next three months. After these three months, absorption of transplanted fat slowed, and the remaining fat was stable throughout the long-term follow-up period. Ecchymosis and hydroncus were observed one week after operation, but neither scars caused by the needle nor sags and crests on the surface of the skin were observed during follow-up. No additional complications or adverse reactions were observed at the 12 month follow-up timepoint. Twelve-month follow-ups were conducted for all 62 patients, and 53 patients subsequently received only one injection in one to six areas. Nine patients received 2 injections in one to three areas, and the injected fat volumes were as follows: 10.0-16.5 mL in the tempora, 2.5-5.0 mL in the geisoma, 10.0-25.0 mL in the frontal part, 0.4-1.0 mL in the palpebra superior, 0.4-1.0 mL in the palpebra inferior, 0.5-2.0 mL in the lacrimal groove, 2.0-4.5 mL in the zygoma (risorius), 8.0-18.5 mL in the cheeks, 2.0-3.5 mL in the nasolabial groove, 3.0-5.0 mL in the chin, 0.8-2.0 mL in the marionette lines, and 4.0-6.0 mL in the submaxilla contour line. Pre- and postoperative photos were used by patients, plastic surgeons, and independent third parties who were otherwise uninvolved with the research to evaluate the effects of the injections on patient appearance. Upon follow-ups 12 months after the second operation, facial soft tissue depression deformities had improved and patients were satisfied; the transplanted fat survived long-term with low levels of absorption and with no infections, fat liquefaction, paresthesia, or other complications. Mean satisfaction rates were higher than 90% in test group patients, but were lower than 70% in control group patients, after 12 and 24 months (Table 2, Figure 7). Complications noted during follow-ups are listed in Table 3. Regardless of initial stage, patients’ skin clearly improved; VISIA and SOFT5.5 revealed that skin texture, elasticity, pore size, and moisture improved, and trends towards improvement were also observed for wrinkles and splashes (Tables 4 and 5).

**Table 2:** Three methods for evaluating satisfaction after 12 and 24 months in nanofat and control group patents (cases, %)

Evaluators	n	I	II	III	V
	12 months				
A	62 vs	50 (80.65)vs	7 (11.29)vs	2 (3.23)vs	3 (4.83)vs
	77	31 (40.26)	21 (27.27)	15 (19.48)	10 (12.99)
B	62vs	54 (87.10)vs	5 (8.06)vs	1 (1.61)vs	2 (3.23)vs
	77	34 (44.16)	23 (29.87)	13 (16.88)	7 (9.09)
C	62vs	48 (77.42)vs	8 (12.90)vs	2 (3.23)vs	4 (6.45)vs
	77	32 (41.56)	19 (24.68)	15 (19.48)	11 (14.28)
	24 months				
A	47vs	38 (80.85)vs	4 (8.51)vs	2 (4.26)vs	3 (6.38)vs
	58	23 (39.66)	16 (27.59)	11 (18.96)	8 (13.79)
B	47vs	40 (85.11)vs	3 (6.38)vs	1 (2.13)vs	3 (6.38)vs
	58	25 (43.10)	17 (29.31)	12 (20.69	4 (6.90)
C	47vs	36 (76.60)vs	6 (12.77)vs	3 (6.38)vs	2 (4.26)vs
	58	23 (39.66)	15 (25.86)	15 (25.86	5 (8.62)

A, patients; B, surgeons; C, entrusted assessment

I, very satisfied; II, satisfied; III, average; IV, unsatisfied

**Table 3:** Postoperative incidence rates in nanofat and control group patients (cases, %)

Stage (post-operative)	Cases	Infection	Cyst	Induration	Scar hyperplasia	Topopares-thesia	Rate
**7 days**	62	3 (4.84)	0 (0)	0 (0)	0 (0)	11 (17.74)	22.58
	77	5 (6.50)	0 (0)	0 (0)	0 (0)	16 (20.78)	27.28
**6 months**	62	0 (0)	0 (0)	0 (0)	0 (0)	0 (0)	0
	77	0 (0)	0 (0)	1 (1.30)	2 (2.60)	0 (0)	3.90
**12 months**	62	0 (0)	1 (1.61)	1 (1.61)	0 (0)	0 (0)	0
	77	0 (0)	0 (0)	1 (1.30)	1 (1.30)	0 (0)	2.60
**24 months**	47	0 (0)	0 (0)	0 (0)	0 (0)	0 (0)	0
	58	0 (0)	0 (0)	1 (1.30)	0 (0)	0 (0)	1.30

**Table 4:** VISIA and SOFT5.5 values of facial skin in nanofat group patients (means ± SD, n=62)

	Before	6 Months	12 Months	24 Months
**VISIA value**				
**Splash**	48.13±11.24	41.17±11.75*	38.79±10.05*	40.78±12.14*
**Wrinkles**	57.32±12.78	31.76±10.38*	32.13±9.42*	33.74±9.77*
**Texture**	54.91±13.05	43.27±13.55*	42.06±12.37*	41.89±11.91*
**Pore**	67.53±16.19	61.79±16.77*	59.05±16.92*	58.87±16.75*
**Sclererythrin**	48.71±9.92	24.65±7.69*	27.54±7.07*	31.26±8.38*
**SOFT5.5 value**				
**pH**	5.73±0.82	5.12±0.69	5.36±0.83	5.43±0.85
**Moisture**	47.87±14.25	78.12±9.38*	72.85±9.31*	68.76±8.92*
**Elasticity**	11.05±4.74	15.31±4.16*	13.87±5.42*	13.22±5.31*
**Grease**	45.82±11.67	30.12±9.14*	36.09±10.12*	39.83±9.79*

*P<0.01 vs. before

**Table 5:** VISIA and SOFT5.5 values of facial skin in control group patients (means ± SD, n=77)

	Before	6 Months	12 Months	24 Months
**VISIA value**				
**Splash**	49.82±12.17	42.05±11.83*	48.87±12.42	49.03±12.59
**Wrinkles**	56.98±13.24	38.45±11.14*	55.81±12.65	58.04±13.85
**Texture**	52.96±12.87	44.23±12.79*	51.68±11.47	53.25±13.11
**Pore**	65.46±15.91	60.06±14.37*	63.95±13.84	67.12±16.08
**Sclererythrin**	46.99±9.35	28.81±7.45*	44.88±9.02	45.75±10.66
**SOFT5.5 value**				
**pH**	5.81±0.91	5.63±0.72	5.75±0.86	5.77±0.89
**Moisture**	49.37±8.40	68.56±11.14*	51.23±9.76	50.25±9.33
**Elasticity**	10.89±4.93	14.89±5.17*	11.02±4.87	10.32±4.45
**Grease**	47.01±10.18	33.42±8.21*	46.76±9.25	45.97±9.38

*P<0.01 vs. before

## DISCUSSION

Autologous fat tissue has been considered one of the best materials for filling soft tissue and is widely used in both reconstructive and cosmetic surgery. However, autologous fat grafts are associated with significant volume retention and cell survival problems, and no consensus has been reached regarding an optimal alternative technique. ASCs are the ideal seed cells for tissue regeneration, and several studies have demonstrated the efficacy of ASCs in antiaging treatments, reconstruction of tissue damage, and promotion of fat transplant survival [21–24]. Previous research on fat transplantation has focused mainly on its application as filling material to improve shape or on enhancing the survival of transplanted fat cells and increasing the quality and duration of the transplants. Mojallal *et al*. found that subcutaneous injections of fat tissue made the skin look younger; survival of transplanted fat tissue resulted not only in volumetric expansion and the formation of new collagen that tightened the skin and made it glossier, but also in the formation of new blood vessels in the transplant area [25]. In addition, fat tissue injections can increase the volume of newly-formed tissue in the transplant area. ASCs in fat tissue foster reparative regeneration; differentiation of ASCs stimulates the production of large amounts of collagen I protein and smaller amounts of collagen V and VI proteins, the regeneration of fibroblasts, and the secretion of large amounts of cellular matrix, all of which together help to repair the original dermal break, reconstruct and rehabilitate skin structure, and eliminate wrinkles. Fat cells lack a reliable blood supply in the early stages after transplantation. Before a dedicated blood supply is established, transplanted fat tissue obtains nutrition mainly via osmosis from surrounding tissue fluid. However, due to its isolation from surrounding tissue, fat tissue at the center of the transplant may die and liquefy as a result of persistent ischemia and hypoxia. Carpaneda and Ribeiro proposed the “borderland” concept and found that 40% of the tissue in the borderland within 1.5±0.5mm from the edge of the transplant survived [26]. Doi *et al*. also found transplant fibrillation and fat cell volumes were lower in center part of the transplant compared to the border, suggesting that transplant absorption occurs mainly in the center of the transplant; it is therefore vital to improve the nutrition and blood supply of the center of the transplant in the early stages of recovery [27, 28]. SVF obtained from liposuction is composed of many types cells, including mature fat cells, ASCs, endothelial cells, fibroblasts, and pericytes; furthermore, ASCs can differentiate to lipoblasts or vascular endothelial cells [29–34]. Recent studies have confirmed that SVF can facilitate the rapid vascularization of fat transplants and in turn increase survival rates [35–38]. Liposuction and the mechanical emulsification method generate nanofat with a diameter of approximately 50-100 μm that can help fat transplants, particularly the cells in the center, connect effectively to the ASCs and cell factors in SVF. However, PRF, or concentrated blood platelets generated from autologous whole blood via centrifugal separation, contains growth factors that foster tissue repair and regeneration, such as platelet-derived growth factor (PDGF), vascular endothelial growth factor (VEGF), transforming growth factor β1/β2 (TGFβ1/TGFβ2), and epidermal growth factor (EGF) and so on. In the clinical setting, autologous PRF has the added advantages of preventing immunologic rejection, simple production, and few complications. The beneficial effects of PRF in damage repair and tissue regeneration have been widely reported [39–40]. Here, we treated 62 patients with facial depression deformities by transplanting a mixture of newly-isolated nanofat, which is rich in SVF, PRF, and autologous fat compared to a control group including 77 patients treated with the traditional autologous fat graft. At the 12-month follow-up timepoint, the transplanted fat of test group had largely survived in good condition, and most patients were satisfied with the results of a single injection. Only 9 patients (14.52%) from test group required a second injection, indicating that graft re-absorption was relatively rare and complications were minimal for these fat transplants. In contrast, traditional autologous fat transplantation requires repeated injections and is associated with higher rates of cyst formation and liquefaction. Furthermore, none of the patients in this study exhibited topoparesthesia, fat liquefaction, induration, or paresthesia, indicating that this safe and remarkably effective method might be the best option for clinical treatment of many types of tissue depression. Our results also indicate that patients’ skin improved more in some regards than in others after treatment. VISIA and SOFT5.5 revealed that skin texture, elasticity, pore size, and moisture clearly improved. A trend towards improvement in wrinkles and splashes was also observed, likely due to the paracrine effects of the SVF in nanofat and the anti-aging effects of cell factors in PRF.

In summary, transplantation of newly-isolated nanofat containing SVF, PRF, and autologous structural fat granules was used to improve facial shape and signs of aging in patients with facial soft tissue depression deformities. Patients expressed a high degree of satisfaction with the results, fat tissue survived long-term in the transplant area, no serious complications were observed, and only one injection was required in most cases. These results suggest that NFSCs function similarly to mesenchymal stem cells and share many of the biological characteristics of traditional fat stem cell cultures isolated via non-collagenase digestion. The safe and remarkably long-lasting method described here might enhance the survival of fat transplants to improve ductility, facial contour refinement, and the appearance of the skin. Additional studies are needed to confirm the benefits of this new method.

## MATERIALS AND METHODS

### Patient consent and ethical approval

This study was approved by the Research Ethics Committee of The Fifth Affiliated Hospital of Guangxi Medical University on 10/08/2014. Sixty-two patients (test group) with asymmetric facial tissue, depression deformity, and aging skin were selected at random for inclusion. Consultations and diagnosis occurred at the Plastic and Aesthetic Surgery department of the Fifth Affiliated Hospital of Guangxi Medical University & The First People’s Hospital of Nanning and at the Nanning Dream Plastic and Aesthetic Hospital between October 2014 and October 2016. Patients were between 24 and 55 years old with an average age of 28.5; 12 patients were men and 50 were women. All patients had skin types of III or IV. Seventy-seven age-, gender-, and symptom-matched control patients were enrolled over the same period and underwent traditional autologous fat transplantations. Pre- and postoperative photos were taken of each patient, and all patients signed informed consent forms.

### Extraction and preservation of platelet-rich fibrin (PRF)

**Figure 8:**
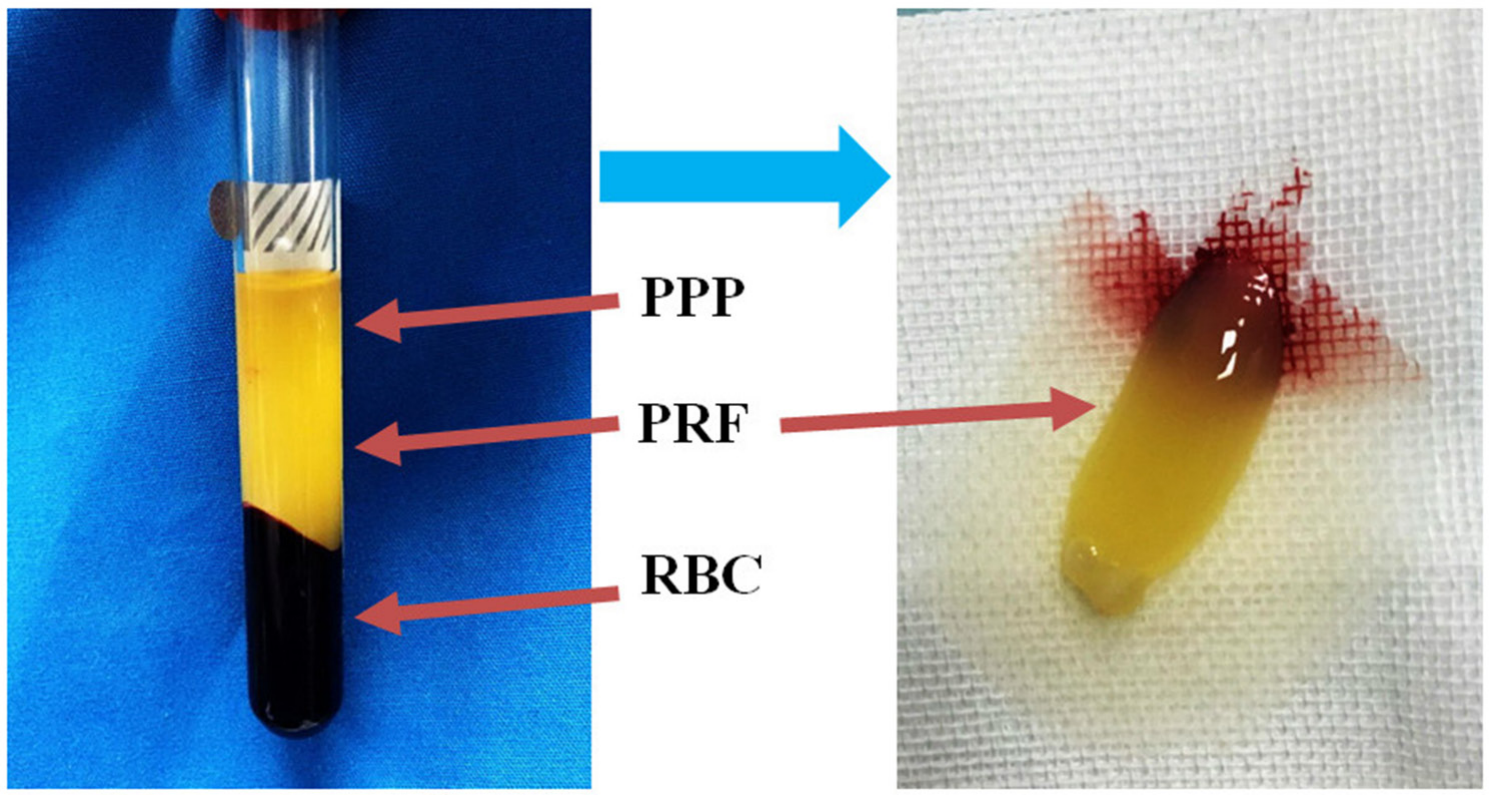
Isolation of PRF clots from venous whole blood via centrifugation After a single centrifugation, red blood cell elements were located in the lower phase, the middle phase containing PRF clots was transferred into a new tube, and the upper phase consisted of platelet-poor plasma (PPP).

PRF was prepared as previously reported [41]. Briefly, 20 mL of venous blood was collected in 4 glass-coated, aseptic, negative-pressure plastic tubes without anticoagulant from some patients during liposuction. After centrifugation at 2700 rpm/min for 12 minutes followed by a 5-minute rest period, the blood was separated into three layers: the top layer, which consisted of transparent yellow liquid, was platelet poor plasma (PPP); the middle layer, which consisted of faint yellow gelatinous matter, was the PRF clots (Figure 8); and the bottom layer, which consisted of rufous matter, was erythrocyte fragments. Superfluous liquid was discharged, PRF was collected, and the fibrous clots was then prepared. The red ends of PRF contained large amounts of cell factor, so they were cut off lengthways to ensure that they were well-distributed. We prepared three different proliferation media (DMEM, 100 mL/L FBS, 100 U/mL penicillin, and 100 μg/mL streptomycin plus 1/10, 2/10,or 3/10 PRF, respectively) and three different adipogenic inductive media (containing nurtient solution, 200 μM indomethacin, 10 μM insulin, 0.5 mM 3-isobutyl-1-methylxanthine, and 1μM dexamethasone plus 1/10, 2/10, or 3/10 PRF, respectively). These media were then stored at 4°C until they were used in the following experiments.

We detect the slow release of growth factors according to the method described by Dohan Ehrenfest *et al*. [42]. Each PRF clots sample was transferred into a sterile tube with 4 mL DMEM for preservation and incubated for 1, 24, 72, or 168 hours. All DMEM samples were then stored at −20°C until enzyme-linked immunosorbent assay (ELISA) quantification was performed. VEGF, PDGF-AB, TGF-β, EGF, IL-6, IGF-1, and MMP-1 levels in the experimental PRF-conditioned DMEM samples were analyzed according to the ELISA kit manufacturer’s protocol.

### Collection of structural fat and nanofat

**Figure 9:**
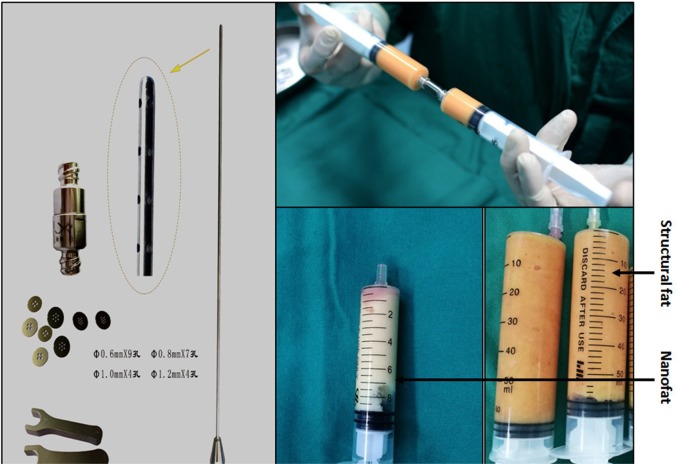
Lipoaspirate was mechanically emulsified after rinsing Emulsification of the fat was achieved by transferring the fat between two 20-cc syringes connected to each other by a female-to-female Luer-Lok connector. After 3 min of continuous transfers, the fat became emulsified. At the end of the fragmentation process, the fat became liquid and took on a whitish appearance.

Fat was collected using the Coleman method with some improvements and involved a low area supply from the inner and outer thigh, low negative pressure suction (20 mL reserved air method), and low speed centrifugation (1000 rpm/min for 2 min). Depending on each patient’s condition, local tumescent infiltration anesthesia was administered to the fat supply area. Liposuction was performed using a 3.5 mm polyporous specific needle starting in deep tissue and moving up repeatedly with a fan-shaped motion. The collected fat granules were prepared by washing with physiological saline (Figure 9). Refined active fat granules were separated into two parts: one was used as seed cells for transplantation (structural fat), and the other was used to make nanofat, which involved briefly mechanically emulsifying the rinsed lipoaspirate. Emulsification was achieved by transferring the fat between two 20-cc syringes connected to each other by a female-to-female Luer-Lok connector. After 3 min of continuous transfer, the fat became an emulsified liquid with a whitish appearance. The emulsified fat was filtered through a superfine filter to obtain nanofat (Figure 9).

### Isolated culture and identification of NFSCs

The nanofat was then centrifuged at 8000 rpm for three minutes and immediately resuspended without collagenase digestion. The enriched SVF cell mass was resuspended with 10% fetal bovine serum in a DMEM nutrient solution. The resuspended cells were then cultured in a dish in a 37°C incubator with 5% CO_2_ to allow NFSCs to proliferate. The nutrient solution in the dish was changed once every 24 hours, and cells that had not attached to the dish were removed. After that, the nutrient solution was changed once every 3 days. NFSCs proliferation was monitored using a microscope. Once third-passage cells reached 80% to 90% confluence, they were resuspended in PBS with 30 mL/L FBS, counted, and transferred into nine 1.5 mL centrifuge tubes. After they were trypsinized using Accutase (Innovative Cell Technologies, San Diego, CA, USA), the P3 NFSCs were analyzed using flow cytometry after one of the following rabbit anti-human primary antibodies was added: CD29-PE, CD44-PE, CD49d-PE, CD54-PE, CD90-PE, or CD105-PE. Incubation with CD34-PE, CD45-PE, CD106-PE, and the negative control IgG-PE antibodies was performed in the dark at room temperature for thirty minutes. The cells were then washed with PBS 3 times, resuspended in PBS with 30 mL/L FBS, and used for flow cytometry to measure the surface markers on NFSCs. To ensure that flow cytometry could detect cell differentiation, adipogenic, osteogenic, and chondrogenic culture media were used to culture the third-passage NFSCs. After two or three weeks, the relative histological detection method was employed to evaluate the differentiation of NFSCs.

### Measurement of PRF-induced growth and proliferation in NFSCs

Third-passage human NFSCs were examined using the CCK-8 method. The cells were transferred to a 96-well plate at a concentration of 1×10^4^ cells/well and cultured for 24 hours in an incubator at 37°C with 50 mL/L CO_2_, after which the nutrient solution was poured off. Cells were then randomly divided into 4 groups with three wells per group (n=3); for the 3 experimental groups, culture medias with PRF concentrations of 1/10, 2/10, or 3/10 were added, while common culture media was added for the control group. After 9 additional days of culture, during which liquid supernatant was removed daily at the same time for each group, 50 μL CCK-8 working fluid was added to each well followed by 4 hours of culture. After 10-minute oscillations, the OD at the 450 nm wavelength was detected using a microplate reader.

### Scanning electron microscopy

After NFSCs and PRF clots were co-cultured for 72 hours, the morphology of cells growing on the PRF clots was evaluated using scanning electron microscopy (SEM, Phenom ProX, Netherlands) images taken on an SEM-FE MIRA II LMU (TESCAN, Brno-Kohoutovice, Czech Republic). Samples were dehydrated and fixed in 2.5% glutaraldehyde, washed in PBS and then in 50, 70, 80, 95, and 100% EtOH, a mixture of EtOH and Hexamethyldisilazane (HMDS) (Sigma, Aldrich), and 100% HMDS solution, and then gold/palladium coated. ImageJ was used for image analysis.

### Measurement of PRF-induced changes in adipogenic differentiation

Third-passage human NFSCs were transferred to 6-well plates at a concentration of 2×10^5^ cells/well and cultured in an incubator at 37°C with 50 mL/L CO_2_, after which the nutrient solution was poured off. Cells were then randomly divided into 4 groups with 3 wells per group (n=3); for the 3 experimental groups, culture medias with PRF concentrations of 1/10, 2/10, or 3/10 were added to the basal adipogenic induction media (containing 200 μM indomethacin, 10 μM insulin, 0.5 mM 3-isobutyl-1-methylxanthine, and 1μM dexamethasone), while the common adipogenic induction media was added for the control group. The liquid in the incubator was changed once every 3 days. After cells in all groups had been cultured for 14 days, RNA was extracted using the Trizol method and cDNA was generated by reverse transcription. Real-time quantitative qPCR was performed according to the manufacturer’s instructions and PPARγ, C/EBPα, and ADD1 mRNA expression were measured. This experiment was repeated 3 separate times. Primer sequences are shown in Table 6.

**Table 6:** Primer sequences used in human NFSCs

Gene (human)	Annealing temperature (°C)	Forward/Reverse	Primer sequence	PCR product size (bp)
**PPARγ2**	57	Forward	AATCAAAGTGGAACCTGCATC	179
		Reverse	TTCGGAAGAAACCCTTGCAT	
**C/EBPα**	59	Forward	AGCCGATATCTTGTATCTAGCCT	136
		Reverse	CTCATTTTGGCAAGTATCTGAGC	
**ADD1**	58	Forward	TATGACCGCAAACGTCCCG	204
		Reverse	ATGAGCTGAGACCACCCG	
**GAPDH**	58	Forward	ATGTTGTCGCCATCAATGATCC	197
		Reverse	GTACTCGGCACCAGCATCAC	

### Transplantation of structural fat combined with PRF and nanofat

Structural fat granules, nanofat, and PRF were mixed at the proper proportions for transplantation into depressed areas with signs of severe aging. A sharp needle was first used to pierce the skin and a blunt needle was then used to inject the transplant under local anesthesia. The syringe plunger was drawn back first to confirm that no blood was present, thus avoiding injections into blood vessels, especially around the eyes. Injections were performed slowly with as little pressure as possible. To avoid cell accumulation, which affects blood transportation, the multipoint and layering method, either steadily in each sector or with striation, was used for injections. A volume 25-30% greater than the desired final amount was injected. After injection, local massages were used to ensure optimal fat granule distribution. Dressings for the implant location were changed every 2 days using light pressure and intravenous antibiotic injections were administered for 3 days to prevent infection.

### Postoperative management and follow-up

The transplant area was immobilized for one week after the operation to minimize the activity of surrounding muscles (masseter, temporalis, etc.) and prevent damage to new blood vessels in the transplanted fat. Photos of patients’ faces and the most preoperatively depressed areas were taken at various postoperative timepoints; photos were used to evaluate facial shape and skin quality before and after the operation. If the depressed area had not improved sufficiently within six months of the first injection, a second injection was performed to further increase local tissue quantity.

Follow-ups occurred 7 days, 3 months, 6 months, and 12 months after operation for all 62 patients and after 24 months for 47 of the patients. Quantitative analyses and evaluations of the skin were performed for all 62 test group and 77(12 months) and 58(24 months) control group patients. All photos used for evaluation were taken by the same doctor with the same camera and illumination source and from the same angle. A VISIA skin image analyzer and a SOFT5.5 skin test instrument were used for quantitative measurements. Detections were conducted at 24°C and 40-50% humidity. The SOFT5.5 skin tester was placed vertically on the skin and pH, moisture, elasticity, and grease level were measured after the reading had stabilized for 5-10 seconds. These measurements were repeated 5 times, and average values were used in analyses. The VISIA skin image analyzer was used to photograph the subjects’ faces while minimizing the effects of reflected light and obstruction by hair as much as possible. The VISIA skin image analyzer automatically generated an absolute score that indicated the strength of the skin in the chosen area for each patient.

### Observation index and curative effect

Observation index accounted for the difference in facial contour and skin texture before and after treatment, complications that occurred, and degree of satisfaction for all patients.

Curative effect was defined as follows: Perfect (very satisfied): natural and smooth facial contour, delicate skin, and obvious improvement in signs of aging after treatment; Good (satisfied): natural and smooth facial contour and obvious improvement in signs of emaciation and aging, but facial expressions are not perfect enough, after treatment; Average (partially satisfied): some improvement in signs of emaciation and aging, but unnatural and less smooth facial contour, after treatment; Poor (unsatisfied): minimal changes in facial appearance after treatment.

### Statistical analysis

Data are shown as means ± standard deviation statistical analyses were performed using SPSS17.0 software. Multi-group measurements were compared using one-way analysis of variance, comparisons between two groups were evaluated using Scheffe’s post-hoc test, and enumerated data were analyzed using the χ^2^ test. *P*<0.05 indicated statistical significance.
